# Comparison of three surgical approaches for thoracolumbar junction (T12-L1) tuberculosis: a multicentre, retrospective study

**DOI:** 10.1186/s12891-019-2891-7

**Published:** 2019-11-09

**Authors:** Yanping Zeng, Peng Cheng, Jiulin Tan, Zhilin Li, Yuan Chen, Li Tao Li, Yonghong Zheng, Gaoju Wang, Jianzhong Xu, Zehua Zhang

**Affiliations:** 1 Department of Orthopaedics, Southwest Hospital, Third Military Medical University, Chongqing, 400038 China; 2 Department of Orthopaedics, Lanzhou Military Region General Hospital, Lanzhou, China; 3 Department of Orthopaedics, Yulin People’s Hospital, Yulin, China; 4 Department of Orthopaedics, 8th Medical Centre of Chinese PLA General Hospital Tuberculosis Research Institute, Beijing, China; 5 Department of Orthopaedics, Xi’an Jiaotong University Second Affiliated Hospital, Xi’an, China; 6 Department of Orthopaedics, Affiliated Hospital of Southwest Medical University, Luzhou, China

**Keywords:** Spinal tuberculosis, Thoracolumbar junction lesion, Surgical treatment, Outcome

## Abstract

**Background:**

The surgical approaches to thoracolumbar junction (T12-L1) tuberculosis were controversial. We aimed to compare the safety and efficacy of three different procedures through a multicentre retrospective study.

**Methods:**

The medical records of thoracolumbar junction tuberculosis patients (*n* = 177) from January 2005 to January 2015 were collected and reviewed. Forty-five patients underwent anterior debridement and instrumented fusion (Group A), 52 underwent anterior combined with posterior debridement and instrumented fusion (Group B) and 80 underwent posterior-only debridement and instrumented fusion (Group C). Patients with neurological deficit were 10 in Group A, 23 in Group B, 36 in Group C. All patients had a standard preoperative and postoperative anti - tuberculous therapy regimen. Clinical outcomes, laboratory indexes and radiological evaluation of the three groups were compared. Operations at each centre were performed by the respective senior medical teams of the six different hospitals.

**Results:**

All three surgical approaches achieved bone fusion and pain relief. Cases with neurological deficits had different degrees of improvement after surgery. The operative time was 330.2 ± 45.4 min, 408.0 ± 54.3 min, 227.9 ± 58.5 min, and the blood loss was 744.0 ± 193.8 ml, 1134.6 ± 328.2 ml, 349.8 ± 289.4 ml in groups A, B and C respectively.

The average loss of correction was 5.5 ± 3.7° in group A, 1.6 ± 1.9° in group B, 1.7 ± 2.2° in group C, and the difference between groups except B vs C were of statistically significant (*P* < 0.05).

**Conclusions:**

For patients with thoracolumbar junction (T12-L1) tuberculosis, the posterior-only procedure is the better than the anterior-only procedure in the correction of kyphosis and maintenance of spinal stability. The posterior-only procedure is recommended because it achieves the same efficacy as combined procedure with shorter operation time, less blood loss and trauma.

## Background

Tuberculosis (TB) has an important influence on human health, especially in non-rich states [1–3]. China has the second largest TB infected population affecting an estimated 2 million people [4, 5]. Spinal TB may lead to spinal instability, kyphotic deformities, and compression of the spinal nerve, and the thoracolumbar junction (T12-L1) is one of the main metastatic site of musculoskeletal system [6, 7]. Though most spinal TB can achieve satisfactory outcome through standard chemotherapy alone, surgical intervention is still recommended for cases with large paraspinal abscess, spinal instability, neurological injury and severe kyphosis [8, 9]. However, the surgical approaches are still controversial among spinal surgeons. Some surgeons prefer the anterior approach for its direct access to the infection foci, that is of benefit to debride and reconstruct [10, 11]. However, persistent maintenance of spinal stability is outside the scope of such procedure [12, 13]. So, some experts recommend anterior debridement combined with posterior instrumentation which achieve excellent clinical results except for some inconvenient complications [14]. In recent years, posterior-only surgery has gained popularity because of its valid debridement, ensure decompression and kyphosis correction with limited trauma, few complications, low cost and short recovery time [15, 16]. To our knowledge, no study was done to compare the therapeutic efficacy between anterior-only, posterior-only and anterior-posterior procedures for mono-segmental spinal TB focusing on the thoracolumbar junction (T12-L1). Furthermore, there is no study comparing the three surgical methods in multiple centres and on large samples. Therefore, we conducted a multicentre retrospective research to observe the safety and efficacy of three procedures of treating thoracolumbar junction(T12-L1) TB and to provide a reference for its surgical treatment.

## Methods

### General information

**Fig. 1:**
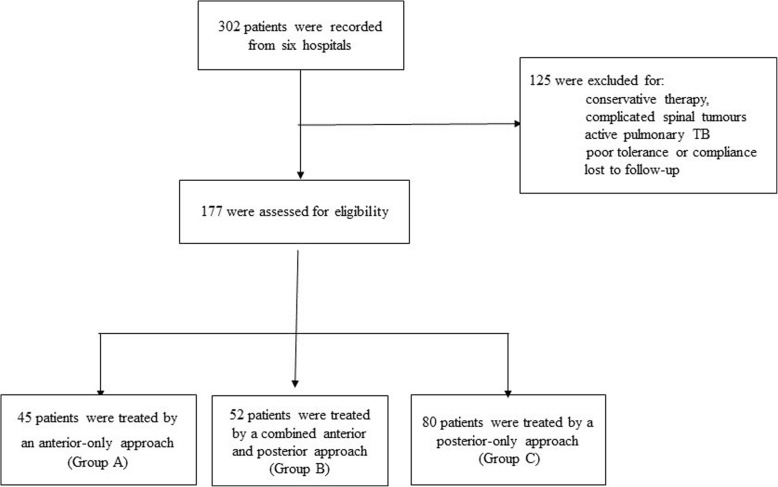
Clinical study design flow diagram

Between January 2005 and January 2015, 302 cases with thoracolumbar junction (T12-L1) TB from six hospitals across China were hospitalized; 125 were excluded because of chemotherapy lonely, poor compliance or tolerance, complicated with active lung TB or spinal tumours, HIV co-infection and lost to follow-up (Fig. 1). The remaining 177 cases were included, comprising 88 males and 89 females with a mean age of 35.2 ± 10.0 years (range 14–62). Forty-five patients were treated by the anterior-only procedure (Group A), 52 by the combined anterior and posterior procedure (Group B) and 80 by the posterior-only procedure (Group C) (Table 1).

**Table 1:** Patients’ Clinical Data

	Group A	Group B	Group C	Statistical Value
Sex (male/female)	21/24	29/23	38/42	
Average age (years)	34.3 ± 10.1	34.4 ± 10.4	35.6 ± 9.9	
Preoperative VAS score	5.7 ± 1.6	6.0 ± 1.9	6.1 ± 1.6	P1 > 0.05/ P2 > 0.05/ P3 > 0.05
Operation time (min)	330.2 ± 45.4	408.0 ± 54.3	227.9 ± 58.5	P1 < 0.05/ P2 < 0.05/ P3 < 0.05
Blood loss (mL)	744.0 ± 193.8	1134.6 ± 328.2	349.8 ± 289.4	P1 < 0.05/ P2 < 0.05/ P3 < 0.05
Final follow-up VAS score	0.6 ± 0.7	0.5 ± 0.6	0.6 ± 0.7	P1 > 0.05/ P2 > 0.05/ P3 > 0.05
Follow-up duration (months)	30.0 ± 7.3	29.7 ± 6.6	28.9 ± 6.1	P1 > 0.05/ P2 > 0.05/ P3 > 0.05

P1:A vs B P2: A vs C P3:B vs C

patients were diagnosed as spinal TB by clinical symptoms, signs, laboratory test, radiological examination and histopathology. Neurological function of the cases was evaluated by American Spinal Injury Association (ASIA) score. Six cases were grade A, 14 were grade C, 47 were grade D and 108 were grade E. The back pain was evaluated by visual analogue scale (VAS) for all patients, and the local kyphotic angle was assessed by Cobb technique.

### Preoperative management

All cases underwent chemotherapy regimens HREZ (rifampicin 450 mg/day, isoniazid 300 mg/day, pyrazinamide 750 mg/day and ethambutol 750 mg/day) for more than 2 weeks preoperatively.

### Operation technique

Operations at each centre were performed by senior surgeons. All cases were treated by general endotracheal anaesthesia, then placed in the appropriate position. (1) In the anterior-only approach, thoracoabdominal procedure was adopted. After the lesion site was completely debrided, the defect area of vertebrae was inserted with a suitable cage or autologous or allograft iliac bone. Then the screw-rods were inserted in lateral anterior of the vertebrae. (2) In the anterior–posterior approach, the prone position was used initially. Dorsal midline incision was performed. The lamina and articular process were exposed, then pedicle screws were implanted in the right places. After correction of the kyphosis angle, bone grafting was performed, and the incision was closed. Then, patients were transferred to the lateral position, and a correctly placed incision was made. The thoracoabdominal approach was used to debride the lesion, decompress spinal cord and graft cage or iliac bone. (3) In the posterior-only approach, the prone position was used. Dorsal midline incision was performed and the lamina and articular process were exposed. After the screws were placed in the right places, the transpedicular space was used to debride lesion tissues, such as abscesses, necrotic discs and endplates. Then, suitable size autograft iliac bone or titanium cage containing cancellous bone was inserted into intervertebral body. At last, installed the rods and rectified the kyphosis and/or scoliosis. Before the surgery of each group was over, isoniazid (0.3 g) and streptomycin (1.0 g) were administered locally, and tubes were placed routinely near the incision.

### Postoperative care

Preventive antibiotic treatment was used within 48 h postoperatively. All cases were advised to use a bracing apparatus till bony fusion. Patients were administered oral HREZ chemotherapy for 6 months after the surgery, then received HRE chemotherapy for 9–12 months. When the drug sensitivity test indicated drug-resistant TB, sensitive drugs would be adjusted. Patients’ ESR rates, liver and kidney function were re-examined regularly. Follow-up was performed at 1, 3, 6, 12 and 18 months, then conducted once each year.

### Statistical analysis

Continuous data were expressed as $\overline{\mathrm{X}}$ ± S.D. The LSD or Dunnett T3 test was used to evaluate differences in operation time, blood loss, kyphosis angle, ESR, VAS score. SPSS version 22 (SPSS, Inc., Chicago, USA) was used for statistical analysis. Values of *P* less than 0.05 were considered to indicate significant differences.

## Results

### General patient characteristics

In group A, the mean patient age, operation time, bleeding and duration of follow-up were 34.3 ± 10.1 years (range 18–62 years), 330.2 ± 45.4 min (range 200–400 min), 744.0 ± 193.8 ml (range 500–1500 mL) and 30.0 ± 7.3 months (range 24–50 months), respectively. In group B, these were 34.4 ± 10.4 years (range 14–61 years), 408.0 ± 54.3 min (range 295–540 min), 1134.6 ± 328.2 ml (range 400–2000 mL) and 29.7 ± 6.6 months (range 24–50 months), respectively. In group C, they were 35.6 ± 9.9 years (range 14–62 years), 227.9 ± 58.5 min (range 123–600 min), 349.8 ± 289.4 ml (range 200–2200 mL) and 28.9 ± 6.1 months (range 24–52 months), respectively (Table 1).

### Laboratory evaluation

The average preoperative ESR values were 34.7 ± 27.0 mm/h (range 2–99 mm/h) in group A, 38.9 ± 30.2 mm/h (range 2 to 99 mm/h) in group B and 36.3 ± 25.0 mm/h (range 2–99 mm/h) in group C. The postoperative ESR values turned to be normal in all cases at 3-month (Table 2).

**Table 2:** Cobb Angle and ESR in Three Groups

	Preoperative Cobb Angle (°)	Postoperative	Final Follow-Up		ESR (mm/h)	
		Cobb Angle (°)	Cobb Angle (°)	Angle Lost (°)	Preoperative	3 Months Postoperative
A	22.7 ± 7.9	11.2 ± 5.4	16.7 ± 7.0	5.5 ± 3.7	34.7 ± 27.0	6.1 ± 4.7
B	18.1 ± 6.8	8.4 ± 4.2	10.1 ± 4.4	1.6 ± 1.9	38.9 ± 30.2	7.4 ± 5.3
C	20.8 ± 8.3	8.7 ± 3.8	10.3 ± 4.0	1.7 ± 2.2	36.3 ± 25.0	7.0 ± 4.5
P1	< 0.05	< 0.05	< 0.05	< 0.05	> 0.05	> 0.05
P2	> 0.05	< 0.05	< 0.05	< 0.05	> 0.05	> 0.05
P3	< 0.05	> 0.05	> 0.05	> 0.05	> 0.05	> 0.05

P1:A vs B P2: A vs C P3:B vs C

### Function scores

Neurologic function scores were tabulated in Table 3. All cases with neurological injury had different degrees of improvement postoperatively. The postoperative VAS of the three groups were decreased significantly at the last follow-up.

**Table 3:** ASIA Classification in Three Groups

ASIA Classification	Group A (n)				Group B (n)				Group C (n)			
	Pre-operative	Post-operative	Final Follow- up	Improve-ment	Pre-operative	Post-operative	Final Follow- up	Improve-ment	Pre-operative	Post-operative	Final Follow- up	Improve-ment
A	0	0	0	0	3	0	0	3	3	0	0	3
B	0	0	0	0	0	0	0	0	0	0	0	0
C	2	0	0	2	6	3	3	3	8	3	2	6
D	8	3	0	6	14	3	1	13	25	8	4	21
E	35	43	45		29	46	48		44	69	74	

Spinal cord function improvement rate: Group A was 80%, Group B was 82.6% and Group C was 83.3%

### Radiological evaluation

**Fig. 2:**
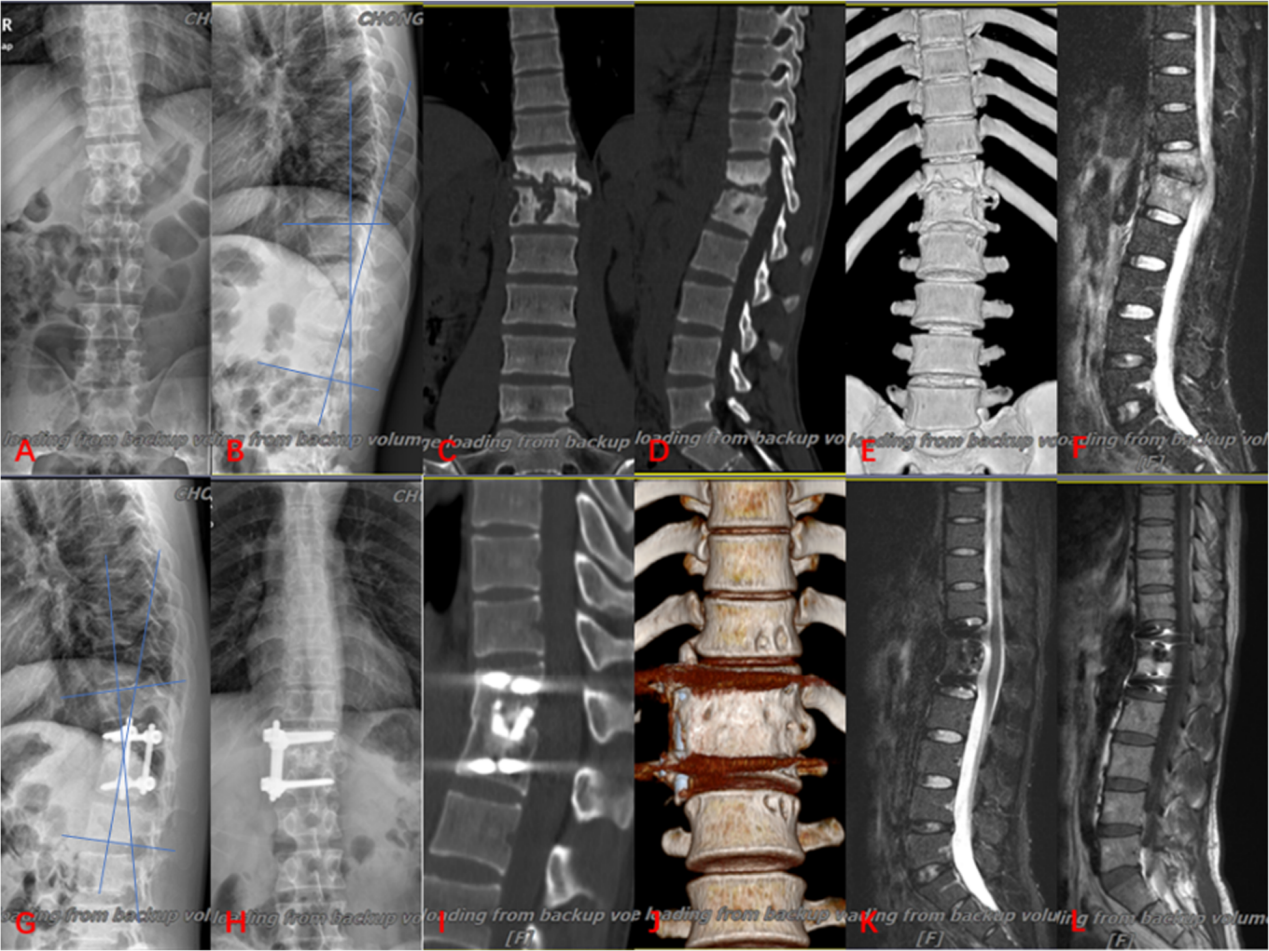
The graph showed a case underwent anterior debridement, bone grafting and screw-rods internal fixation. 25-year-old man with thoracolumbar junction (T12-L1) TB (**a**, **b**) preoperative anteroposterior and lateral X-rays; **c**, **d** preoperative computed tomography (CT); **e** preoperative 3D reconstruction of CT; **f** preoperative MRI; **g**, **h**) X-ray at 18-month postoperative; **i** CT at 24-month postoperative; **j** 3D reconstruction of CT at 24-month postoperative; **k**, **l** MRI at 18-month postoperative

**Fig. 3:**
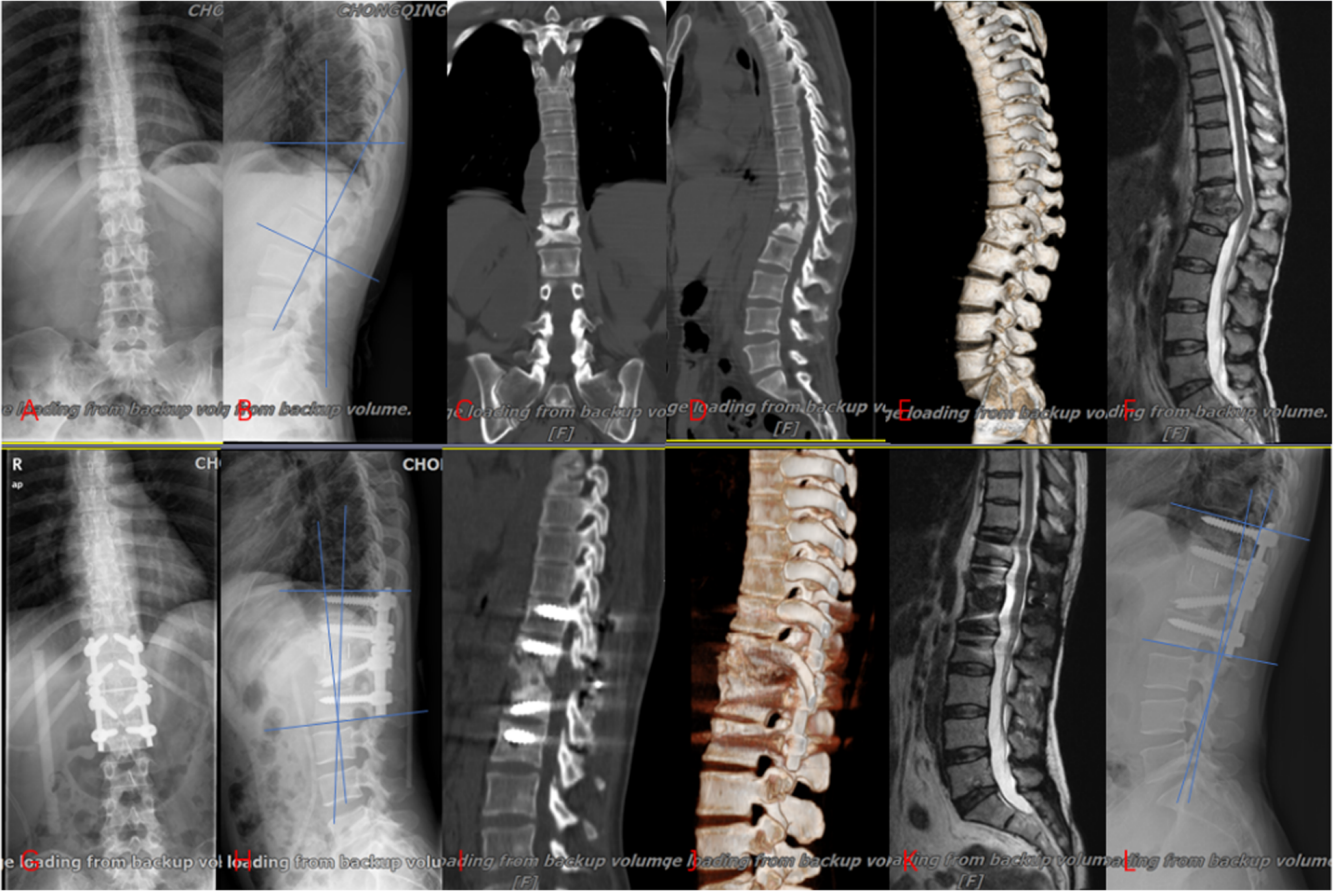
The graph showed a case underwent one-stage anterior debridement, decompression, bone grafting and posterior instrumentation. 38-year-old woman with thoracolumbar junction (T12-L1) TB (**a**, **b**) preoperative anteroposterior and lateral X-rays; **c**, **d** preoperative CT; **e** preoperative 3D reconstruction of CT; **f** preoperative MRI; **g**, **h** X-ray at 1-month postoperative; **i** CT at 6-month postoperative; **j** 3D reconstruction of CT at 6-month postoperative; **k** MRI at 13-month postoperative; **l** lateral X-rays at 56-month postoperative

**Fig. 4:**
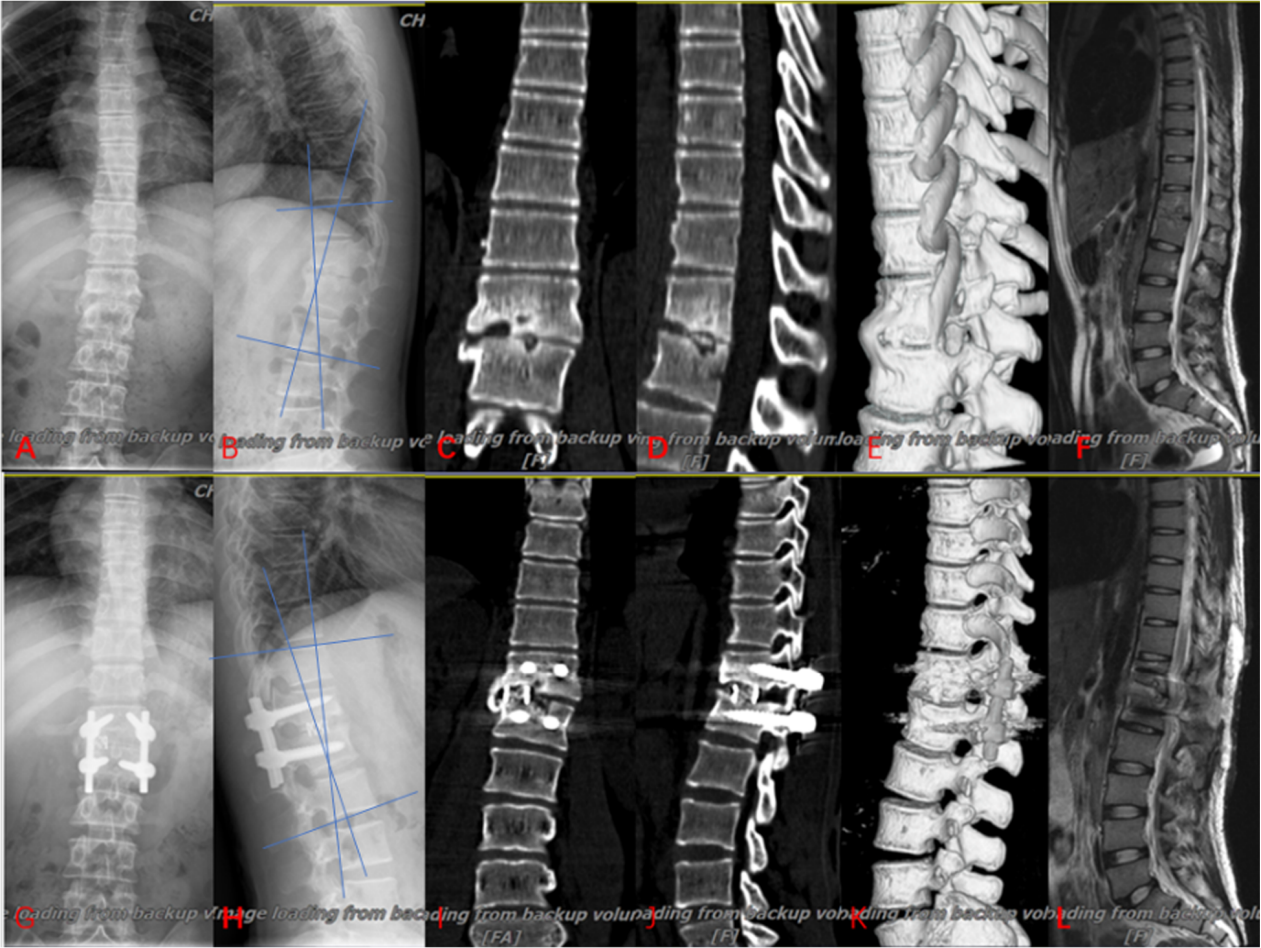
The graph showed a case underwent posterior debridement, decompression, bone grafting and internal fixation instrumentation. 18-year-old man with thoracolumbar junction (T12-L1) TB (**a**, **b**) preoperative anteroposterior and lateral X-rays; **c**, **d** CT preoperative; **e** 3D reconstruction of CT preoperative; **f** preoperative MRI; **g**, **h** X-ray at 1-month postoperative; **i**, **j** CT at 3-month postoperative; **k** 3D reconstruction of CT at 12-month postoperative; **l** MRI at 12-month postoperative

The preoperative mean Cobb angle was 22.7 ± 7.9° in group A (Fig. 2), 18.1 ± 6.8° in group B (Fig. 3) and 20.8 ± 8.3° in group C (Fig. 4). The postoperative Cobb angle decreased significantly to 11.2 ± 5.4° in group A, 8.4 ± 4.2° in group B and 8.7 ± 3.8° in group C. At the last follow-up, the kyphosis angle was 16.7 ± 7.0°, 10.1 ± 4.4°, 10.3 ± 4.0°, in groups A, B and C respectively. Compared with the preoperative Cobb angle, the postoperative and last follow-up Cobb angle in three groups had improved significantly (Table 2). By comparison of kyphosis angle loss, the results showed that the anterior–posterior and posterior-only procedure were superior to the anterior-only procedure in maintaining a corrective effect.

### Complications

In group A, there was 1 case of superficial wound infection, 1 case of cerebrospinal fluid leakage and 1 case of electrolyte imbalance. In group B, there were 1 case of pectoralgia, 1 case of urinary infection, 3 cases of cerebrospinal fluid leakage and 5 cases of electrolyte imbalance. In group C, there were 1 case of superficial wound infection, 5 cases of cerebrospinal fluid leakage, 1 case of electrolyte imbalance and 1 case of refractory intercostal neuralgia. All of these complications were treated successfully or relieved after symptomatic treatment (Table 4).

**Table 4:** Complications related to surgery

Complications	Group A	Group B	Group C
Superficial wound infection	1	0	1
Cerebrospinal fluid leakage	1	3	5
Electrolyte imbalance	0	5	1
Urinary infection	0	1	0
Pectoralgia	0	1	0
Refractory intercostal neuralgia	0	0	1

## Discussion

The thoracolumbar junction (T12-L1) is one of the main sites of metastatic musculoskeletal TB [6, 7]. Although standard anti-TB chemotherapy is the fundamental method of treating spinal TB, suitable and timely surgical intervention for thoracolumbar spinal TB patients can improve spinal stability, decompress the spinal cord and prevent further development of spinal deformity and paralysis or death [17].

The thoracolumbar junction (T12–L1) is sandwiched between the peritoneum and pleura, and various surgical procedures have been used to access the area: anterior-only, anterior–posterior and posterior-only. The most common lesion area is major in anterior column of the spine involving only one motion segment [18]. Therefore, early scholars thought the anterior approach [9], which can allow direct access to the focus, complete debridement and valid decompression, would be the first choice for decompression and debridement in spinal TB. However, it can’t prevent or correct kyphosis deformity to any appreciable extent [19]. In our study, the degree of kyphosis correction after surgery was similar in group A to that in groups B and C, yet Cobb angle losses were larger in group A than in groups B and C. Anterior instrumentation in spinal TB is becoming increasingly popular, because a bone grafting alone does not provide reliable stability. It can be very effective at correcting a deformity and maintaining the correction [20]. The use of biomaterials in lesion area is still in debate as it may undermine efforts to eradicate the infection [21]. However, some experts concluded that the usage of implants are safe because the M. tuberculosis are dividing too slowly to produce strong adhesion or thick biofilm in most cases [22].

The anterior–posterior procedure is an advanced surgical technique that not only achieves radical debridement of the abscess and adequate decompression of spinal cord, satisfactory correction of kyphosis deformity and long-term maintenance spinal stability, but also separates the debridement area from the instrumentation area that can decrease the spread of TB [20]. Disadvantages of the combined approach are longer operation time, higher complication rate, more blood loss and serious trauma. In our study, the operation time, bleeding, and complication rate were much greater with this approach than with other approaches.

Advantages of posterior-only procedure include less blood loss and shorter hospitalization and operative time. Other advantages are adequate decompression of spinal cord, correction of spinal deformity, reconstruction of spinal stability and improvement of patients’ quality of life. Posterior-only procedure may be better in cases with less involved anterior column, which is almost always achieving spontaneous fusion [23, 24]. However, there is a possible risk of TB spreading to posterior healthy area, resulting in infection diffusion and/or fistulas [25]. In this research, the operation time, bleeding and complication rate were less than other groups, and group C achieved the same satisfactory kyphosis correction as group B during the follow-up period.

Our research has some limitations. First, this study was a retrospective rather than a prospective cohort study. Second, operations at each centre were performed by the respective senior medical teams of the 6 different centres, that may result in a certain degree of bias because of differences in their surgical proficiency.

## Conclusions

This multicentre retrospective study showed that the posterior-only approach can be an effective treatment method for thoracolumbar junction (T12-L1) TB patients, with good neurologic recovery, avoidance of kyphosis progression and few complications.

## Data Availability

The datasets used and analysed in this study are available from the corresponding author on reasonable request.

## Abbreviations

ASIA: American Spinal Injury Association
ESR: Erythrocyte sedimentation rate
HREZ: Isoniazid, rifampicin, ethambutol, pyrazinamide
TB: Tuberculosis
VAS: Visual analogue score

## References

[1] Ding P, Li X, Jia Z, Lu Z (2017). Multidrug-resistant tuberculosis (MDR-TB) disease burden in China: a systematic review and spatio-temporal analysis. BMC Infect Dis.

[2] Zumla A, George A, Sharma V, Herbert N, Baroness Masham of I (2013). WHO’s 2013 global report on tuberculosis: successes, threats, and opportunities. Lancet.

[3] Zhao Y, Xu S, Wang L, Chin DP, Wang S, Jiang G, Xia H, Zhou Y, Li Q, Qu X, Pang Y, Song Y, Zhao B, Zhang H, He G, Guo J, Wang Y (2012). National survey of drug-resistant tuberculosis in China. N Engl J Med.

[4] Yao Y, Song W, Wang K, Ma B, Liu H, Zheng W, Tang Y, Zhou Y (2017). Features of 921 patients with spinal tuberculosis: a 16-year investigation of a general Hospital in Southwest China. Orthopedics.

[5] Boachie-Adjei O, Papadopoulos EC, Pellise F, Cunningham ME, Perez-Grueso FS, Gupta M, Lonner B, Paonessa K, King A, Sacramento C, Kim HJ, Mendelow M, Yazici M (2013). Late treatment of tuberculosis-associated kyphosis: literature review and experience from a SRS-GOP site. Eur spine J.

[6] Nagashima H, Yamane K, Nishi T, Nanjo Y, Teshima R (2010). Recent trends in spinal infections: retrospective analysis of patients treated during the past 50 years. Int Orthop.

[7] Jain AK, Dhammi IK, Jain S, Kumar J (2010). Simultaneously anterior decompression and posterior instrumentation by extrapleural retroperitoneal approach in thoracolumbar lesions. Indian J Orthop.

[8] Zhang Z, Luo F, Zhou Q, Dai F, Sun D, Xu J (2016). The outcomes of chemotherapy only treatment on mild spinal tuberculosis. J Orthop Surg Res.

[9] Tuli SM (2007). Tuberculosis of the spine: a historical review. Clin Orthop Relat Res.

[10] Jain AK, Dhammi IK, Prashad B, Sinha S, Mishra P (2008). Simultaneous anterior decompression and posterior instrumentation of the tuberculous spine using an anterolateral extrapleural approach. J Bone Joint Surg Br.

[11] Benli IT, Kaya A, Acaroglu E (2007). Anterior instrumentation in tuberculous spondylitis: is it effective and safe?. Clin Orthop Relat Res.

[12] Soultanis Konstantinos, Mantelos George, Pagiatakis Alexandros, Soucacos Panayotis N. (2003). Late Infection in Patients With Scoliosis Treated With Spinal Instrumentation. Clinical Orthopaedics and Related Research.

[13] Cui X, Li LT, Ma YZ (2016). Anterior and Posterior Instrumentation with Different Debridement and Grafting Procedures for Multi-Level Contiguous Thoracic Spinal Tuberculosis. Orthop Surg.

[14] Zhang HQ, Guo CF, Xiao XG, Long WR, Deng ZS, Chen J (2007). One-stage surgical management for multilevel tuberculous spondylitis of the upper thoracic region by anterior decompression, strut autografting, posterior instrumentation, and fusion. J Spinal Disord Tech.

[15] D’Souza AR, Mohapatra B, Bansal ML, Das K (2017). Role of posterior stabilization and Transpedicular decompression in the treatment of thoracic and thoracolumbar TB: a retrospective evaluation. Clin Spine Surg.

[16] Hu X, Zhang H, Yin X, Chen Y, Yu H, Zhou Z (2016). One-stage posterior focus debridement, fusion, and instrumentation in the surgical treatment of lumbar spinal tuberculosis with kyphosis in children. Childs Nerv Syst.

[17] He Z, Tang K, Gui F, Zhang Y, Zhong W, Quan Z (2019). Comparative analysis of the efficacy of a transverse process bone graft with other bone grafts in the treatment of single- segment thoracic spinal tuberculosis. J Orthop Surg Res.

[18] Varatharajah S, Charles YP, Buy X, Walter A, Steib JP (2014). Update on the surgical management of Pott’s disease. Orthopaedics and Traumatology: Surgery and Research.

[19] Lu G, Wang B, Li J, Liu W, Cheng I (2012). Anterior debridement and reconstruction via thoracoscopy-assisted mini-open approach for the treatment of thoracic spinal tuberculosis: minimum 5-year follow-up. Eur Spine J.

[20] Chen Wen-Jer, Wu Chi-Chuan, Jung Chi-Hsiung, Chen Lih-Huei, Niu Chi-Chien, Lai Po-Liang (2002). Combined Anterior and Posterior Surgeries in the Treatment of Spinal Tuberculous Spondylitis. Clinical Orthopaedics and Related Research.

[21] Faraj AA, Webb JK (2000). Spinal instrumentation for primary pyogenic infection report of 31 patients. Acta Orthop Belg.

[22] Singh K, Vaccaro AR, Kim J, Lorenz EP, Lim TH, An HS (2003). Biomechanical comparison of cervical spine reconstructive techniques after a multilevel corpectomy of the cervical spine. Spine (Phila pa 1976).

[23] Hee HT, Majd ME, Holt RT, Pienkowski D (2002). Better treatment of vertebral osteomyelitis using posterior stabilization and titanium mesh cages. J Spinal Disord Tech.

[24] Qian J, Rijiepu A, Zhu B, Tian D, Chen L, Jing J (2016). Outcomes of radical debridement versus no debridement for the treatment of thoracic and lumbar spinal tuberculosis. Int Orthop.

[25] Zhang H, Sheng B, Tang M, Guo C, Liu S, Huang S, Gao Q, Liu J, Wu J (2013). One-stage surgical treatment for upper thoracic spinal tuberculosis by internal fixation, debridement, and combined interbody and posterior fusion via posterior-only approach. Eur Spine J.

